# Quantification of hand muscle volume and composition in patients with rheumatoid arthritis, psoriatic arthritis and psoriasis

**DOI:** 10.1186/s12891-020-03194-5

**Published:** 2020-04-02

**Authors:** Andreas Friedberger, Camille Figueiredo, Alexandra Grimm, Isabelle d’Oliveira, Tobias Bäuerle, Jürgen Rech, Arnd Kleyer, David Simon, Michael Uder, Georg Schett, Klaus Engelke

**Affiliations:** 1 Institute of Medical Physics, University of Erlangen-Nuremberg, Henkestraße 91, 91052 Erlangen, Germany; 2 FAU Erlangen-Nuremberg, Department of Internal Medicine 3, and Universitätsklinikum, Erlangen, Germany; 3 FAU Erlangen-Nuremberg, Radiological Institute, and Universitätsklinikum, Erlangen, Germany

**Keywords:** Psoriasis, Psoriatic arthritis, Rheumatoid arthritis, Magnetic resonance imaging, Random forest based segmentation, Hand muscle, Fat

## Abstract

**Background:**

Psoriasis (Pso), psoriatic arthritis (PsA) and rheumatoid arthritis (RA) are inflammatory diseases. PsA and RA are characterized by bone and muscle loss. In RA, bone loss has been extensively characterized, but muscle loss has, to the best of our knowledge, not been quantified to date.

**Methods:**

A random forest based segmentation method was used to analyze hand muscle volume in T1 weighted MRI images of 330 patients suffering from Pso, PsA or RA. In addition, fat volume was quantified using MRI Dixon sequences in a small subset (*n* = 32).

**Results:**

Males had a higher relative muscle volume than females (14% for Pso, 11% for PsA, n.s. for RA). Between 40 and 80 years male Pso patients lost 13%, male PsA patients 16%, male RA patients 23% and female PsA patients 30% of their relative muscle volume. After adjustment for age, relative muscle volume in males RA patients was 16% and in female RA patients 9% lower than in Pso patients. In male RA patients relative muscle volume was 13% lower in than in male PsA patients. There was no difference in females. A significant negative correlation (R^2^ = 0.18) between relative intramuscular fat content relative hand muscle volume was observed.

**Conclusion:**

These preliminary data showed that relative hand muscle volume significantly decreased with age in male and female patients with Pso, PsA and RA patients. Independent of age, relative hand muscle volume was significantly smaller in patients with RA compared to the patients with Pso and the difference was twice as large in males compared to females. Also in male but not in female RA patients relative hand muscle volume was significantly smaller than in PsA patients.

## Background

Rheumatoid arthritis (RA) is a chronic inflammatory disease, which is associated with bone and cartilage loss [1]. About two thirds of RA patients also suffer from rheumatoid cachexia (RC) [1], an accelerated involuntary loss of fat-free skeletal muscle mass, which is larger than the decrease related to ‘normal’ aging (sarcopenia). The term RC has already been coined in 1992 [2], but etiology and pathophysiology of RC are still not well understood. RC is underdiagnosed and undertreated [3–5].

Structural bone damage of RA has been assessed using radiographs, magnetic resonance imaging (MRI) and more recently high resolution peripheral quantitative computed tomography (HR-pQCT). In contrast, techniques to quantify muscle properties are still under development. For the diagnosis of RC, advanced muscle and fat imaging is required. 85% of RA subjects have normal BMI [6] because the muscle loss is often compensated by fatty infiltration, resulting in normal body weight. Thus, the sole use of BMI for the diagnosis of RC is misleading.

It has been suggested [7, 8] that the increased fatty infiltration may be one factor to explain the discrepancy of age related decreases between muscle mass and volume in healthy subjects [9]. In addition, adipose tissue is a source of pro-inflammatory cytokines, which triggers inflammatory responses [10, 11], an important observation with potentially high clinical relevance not only in RA but also in psoriatic arthritis (PsA) and psoriasis (Pso). Similar to RA, PsA is also characterized by inflammation of the synovial tissue, which ultimately results in bone, cartilage and muscle damage. However, the production and secretion of pro-inflammatory cytokines is lower than in RA [12, 13], which may explain why PsA is usually less destructive. Pso mainly affects the skin, but subclinical joint inflammation has also been reported [14, 15]. Nevertheless, to the best of our knowledge, no major impact of Pso on muscle has been reported so far.

This study specifically addresses the assessment of hand muscle volume and composition in patients with rheumatoid, psoriatic arthritis and psoriasis using standard clinical and Dixon MRI sequences. The primary study aim was to compare hand muscle volume among the three diseases, independent of age and BMI. Additionally, preliminary results of a hand fat volume assessment were included. For the purpose of this study, psoriasis patients were used as controls.

## Methods

### Patient details

In this study we used existing MRI scans of the dominant hand of 330 ambulatory care patients diagnosed with RA, PsA or Pso, from the Department of Medicine 3 of the University of Erlangen-Nuremberg. Apart from the disease there were no additional inclusion or exclusion criteria. BMI data was available from 206 patients. This subset will be denoted as S_BMI_.

### MR imaging

Routine MRI Scans were performed with a 1.5-Tesla MRI system (MAGNETOM Aera, Siemens Healthcare GmbH, Erlangen, Germany). Subjects were positioned in prone position with head first. For signal reception, a hand/wrist radiofrequency 16-channel coil was used. The standard clinical MR protocol included several sequences from which a fat suppressed T_1w_ TSE was selected for analysis. More recently, a T_2w_ Dixon TSE sequence has been added to the clinical protocol. The specifications of the two sequences used for this study are summarized in Table 1.

**Table 1:** Specifications of the T1 and the Dixon sequences as used in this study. TSE stands for turbo spin echo, TR for the repetition time, TE for the echo time and TD for the time difference between spin and gradient echo formation

Sequence	In-plane matrix		Slices		TR (ms)	TE (ms)	TD (ms)
	Voxel count	Resolution(mm^2^)	Count	Thickness(mm)			
T_1w_ TSE	320 × 320	0.5 × 0.5	64	3.0	760	13	0
T_2w_ Dixon TSE	320 × 320	0.5 × 0.5	30	3.0	3040	78	0

### Image processing

The image processing consisted of pre-processing of the T1 scans, segmentation using the T1 scans and transformation of the segmentation results to the Dixon fat fraction scans.

**Fig. 1:**
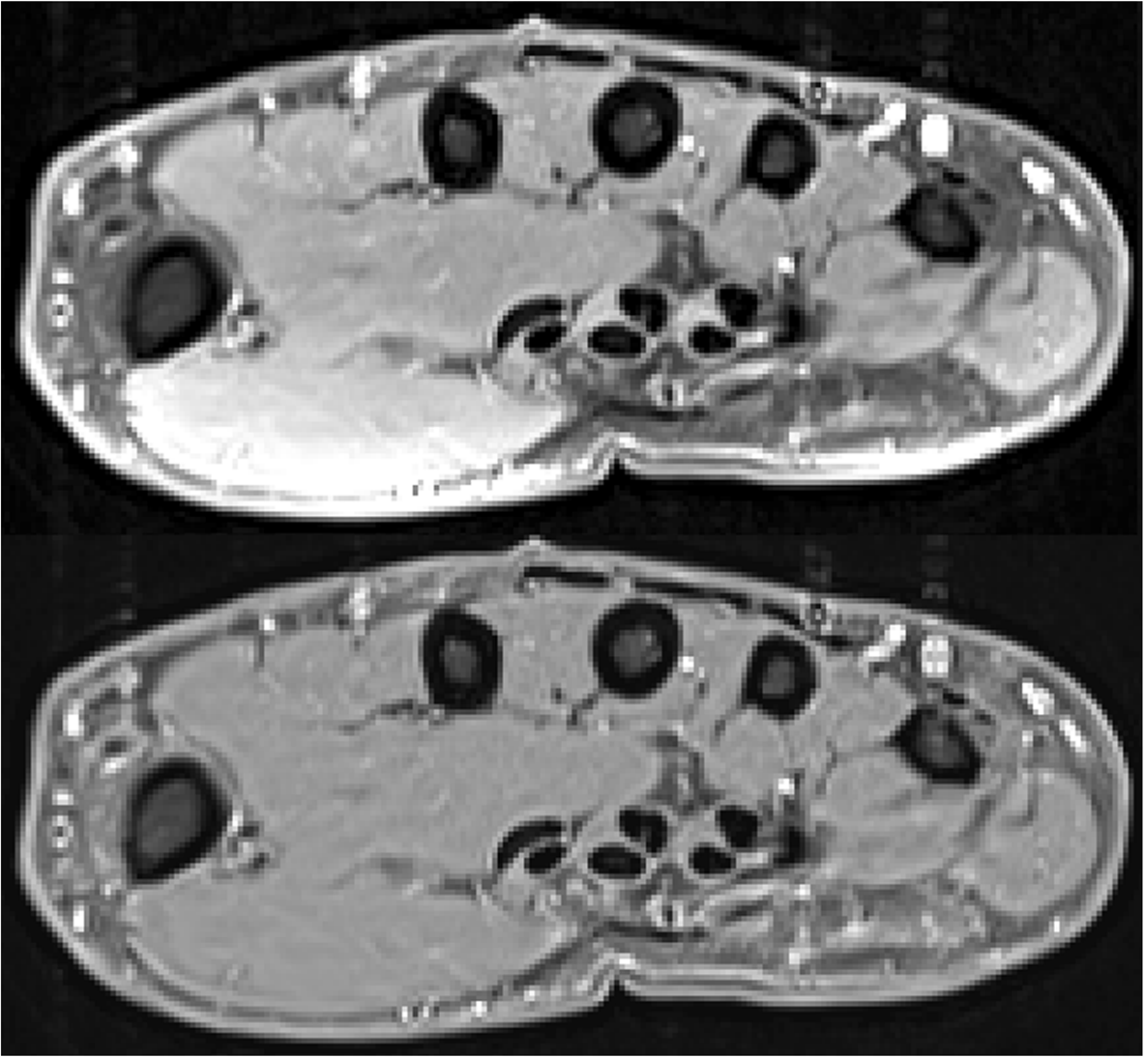
Top: Axial slice of a T1 weighted fat suppressed MRI hand scan in the metacarpal region. Notice the inhomogeneous intensity distribution of the grey values caused by a bias field, especially in the thenar region (lower left). Bottom: Same slice but with removed bias field by non-uniform intensity normalization (N4ITK)

The T1 weighted MR scans were pre-processed with the N4ITK algorithm [16, 17] to remove artificial intensity modulations caused by inhomogeneous magnetic fields (Fig. 1). The segmentation of the hand and of hand muscles was performed in these homogenized images. This step required knowledge of the hand cross sectional area (CSA), which was obtained as a series of 2D contours determined for each slice by thresholding and other basic image processing steps.

**Fig. 2:**
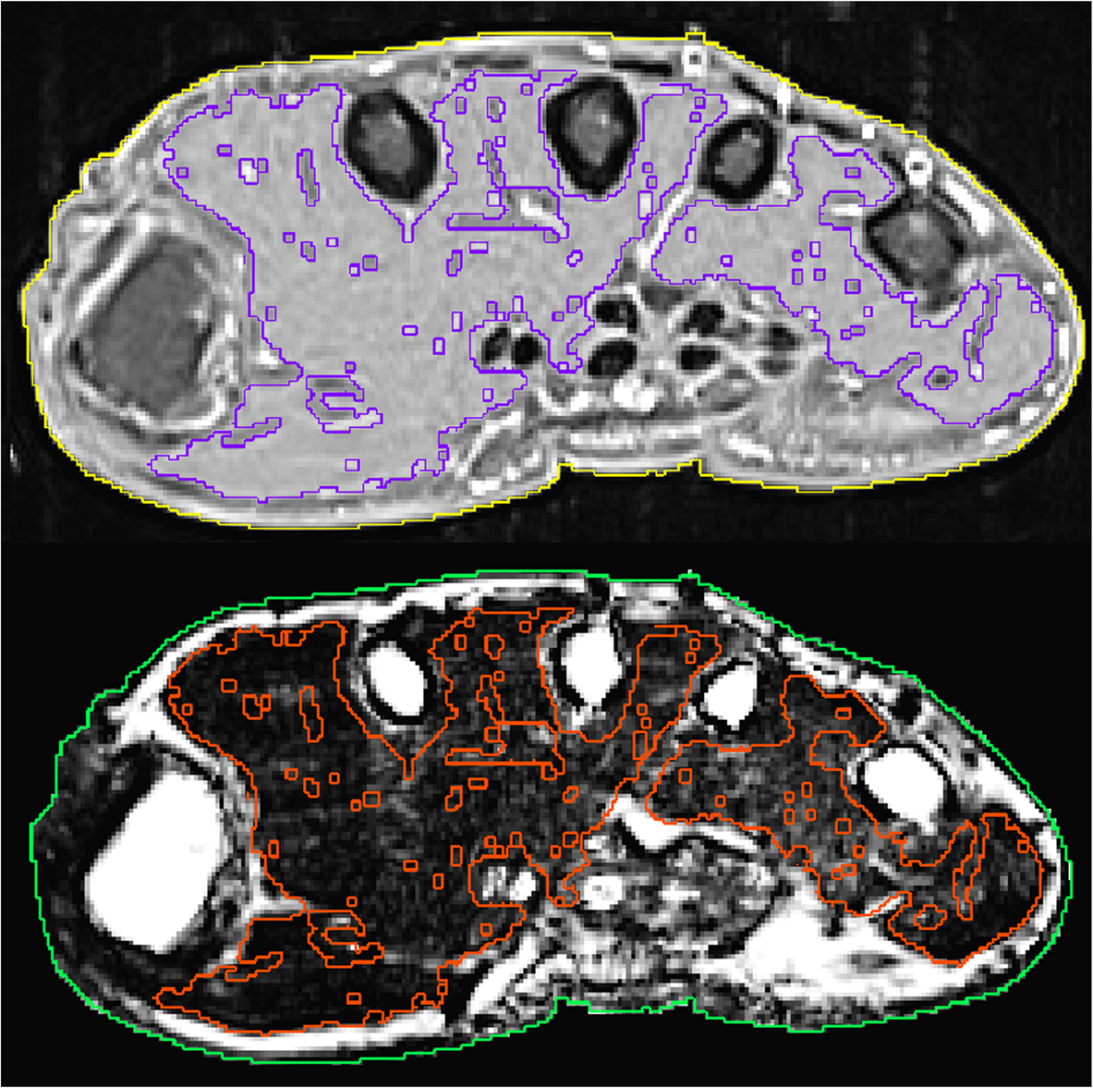
Top: Axial slice with segmented cross sectional area (yellow) and muscle (purple). Bottom: The green CSA and the red muscle segmentation were imported from the processed T1 image via multimodal image registration

The core of the muscle segmentation was a random forest classifier. Random forest is a well-known ensemble learning method from machine learning [18], which is widely used for image segmentation [19]. The random forest classifies each voxel into muscle or background based on image features in the voxel neighborhood. The combination of all voxels classified as muscle constitutes the muscle volume of interest (VOI). The classifier has to be trained beforehand using manually segmented hand scans. The final segmentation step was a Gaussian fit of the histogram of the muscle VOI. Voxels outside the range of the mean ± 2 standard deviations were removed. This removed erroneously included hypointense ligaments and tendons or hyperintense vessels. The results were manually edited by the operator, if necessary. The top panel in Fig. 2 shows a typical segmentation result.

For the quantification of muscle fat content, a 2 pt-Dixon technique was used [20]. This sequence produces a water I_water_ and a fat image I_fat_, from which a quantitative fat fraction image I_ff_ can be calculated:

$$ {I}_{ff}=\frac{I_{fat}}{I_{water}+{I}_{fat}}\ast 1000. $$

I_ff_ assigns a percentage of fat (Fig. 2 bottom) to each voxel. In I_ff_, grey values range from 0 (meaning the voxel consists of 0% fat) to 1000 (meaning the voxel consists of 100% fat), i.e. 1 grey value equals 0.1% fat. In order to restrict the fat analysis to muscle, the muscle VOI determined in the T1 dataset was transferred to I_ff_. For this purpose, the hand surface was registered from the T1 dataset to I_ff_ via rigid multimodal image registration. The resulting transformation was then applied to the muscle VOI (Fig. 2 bottom).

### Output parameters

**Fig. 3:**
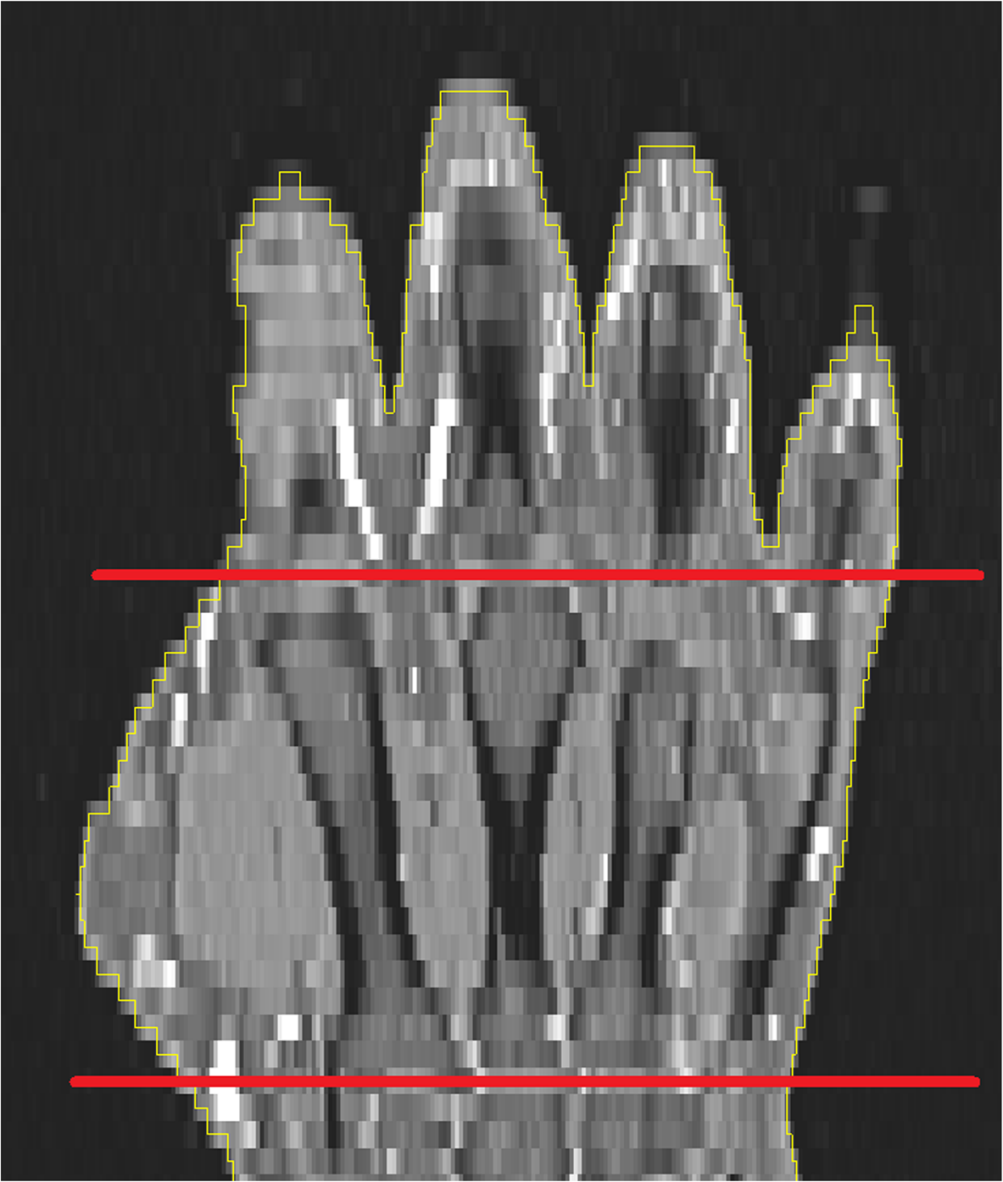
Coronal slice of a hand MR with segmented CSA (yellow). MCP II to MCP IV are clearly visible. Proximal and distal boundaries of MCP III are marked in red, the analysis is limited to the region in between

Direct output parameters calculated from the T1 images were hand (V_H_) and muscle volume (V_M_), derived between user defined proximal and distal ends of the metacarpal bone III (MCP; Fig. 3). These were the accumulated volumes of all voxels participating in the hand resp. muscle segmentation. From these two parameters the relative muscle volume (V_M_^rel^ = V_M_ / V_H_) was calculated. Output parameters from I_ff_ were absolute fat volume (V_F_) and relative fat content (V_f_^rel^ = V_F_ / V_M_) within the muscle VOI. V_F_ was the accumulated volume of all voxels participating in the hand muscle segmentation times their individual fat fraction values I_ff_.

The segmentation reliability was obtained as reanalysis precision errors. Three operators analyzed 14 randomly chosen data sets once (inter-operator) and one operator analyzed these 14 data sets three times (intra-operator). Reanalysis precision errors were calculated as root mean square average of standard deviation and coefficient of variation of individual data sets [21].

### Statistics

In order to evaluate the dependence of hand and relative muscle volume on age and BMI, a linear regression with age and BMI as independent variables was used. For the assessment of differences of V_H_ and V_M_^rel^ among the three groups corresponding to patients with RA, PsA or Pso, the factor disease was added as independent categorical variable to the particular linear model. Group differences, denoted as Δ, were the average difference over the specified age intervals. Since some studies showed an association between hand grip strength and age^3^ [22], age was substituted by age^3^ in a separate linear regression model, under the assumption that hand muscle volume is correlated to hand grip strength.

For a more age-sensitive group difference assessment, the relative muscle volume was partitioned into 10 year intervals from the 5th to the 8th decade. For each decade, group differences were tested using 1-way Anova and post-hoc Tuskey HSD. Further, linear regressions with independent variables age, BMI and hand and relative muscle volume were performed for V_F_ and V_F_^rel^. Since the number of patients, for which fat data were available, was small (*n* = 32), the fat analysis was not stratified for sex or disease.

*p* values below 0.05 were considered significant. All statistics were performed with the statistics software R (version 3.2.5, [23]).

## Results

Patient age is summarized in Table 2. Dixon imaging based fat fraction measurements were available from 32 patients, since just recently added to the clinical protocol. This subset will be denoted as S_dixon_. Inter- and intra-reanalysis precision was excellent (Table 3).

**Table 2:** Age, sex and diagnosis distribution of the cohort

	Pso (*n* = 101)		PsA (*n* = 137)		RA (*n* = 92)	
	male	female	male	female	male	female
n	63	38	61	76	41	51
Age (y)	48 ± 12	54 ± 15	56 ± 14	56 ± 11	62 ± 12	60 ± 14

**Table 3:** Reanalysis precision of hand and muscle volume (measured in the T1 image) and of muscle fat content and ratio (measured in the Dixon images) Precision are shown as root mean square average of the standard deviation displayed in the unit of the variable and of the coefficient of variation displayed in %

	Hand volume		Muscle volume		Fat content		Fat fraction	
Interoperator	5.0 mm^3^	0.19%	1.5 mm^3^	0.24%	364.4 mm^3^	0.60%	0.09%	0.07%
Intraoperator	3.4 mm^3^	0.13%	0.35 mm^3^	0.05%	41.6 mm^3^	0.10%	0.006%	0.04%

### Assessment of hand volume

**Fig. 4:**
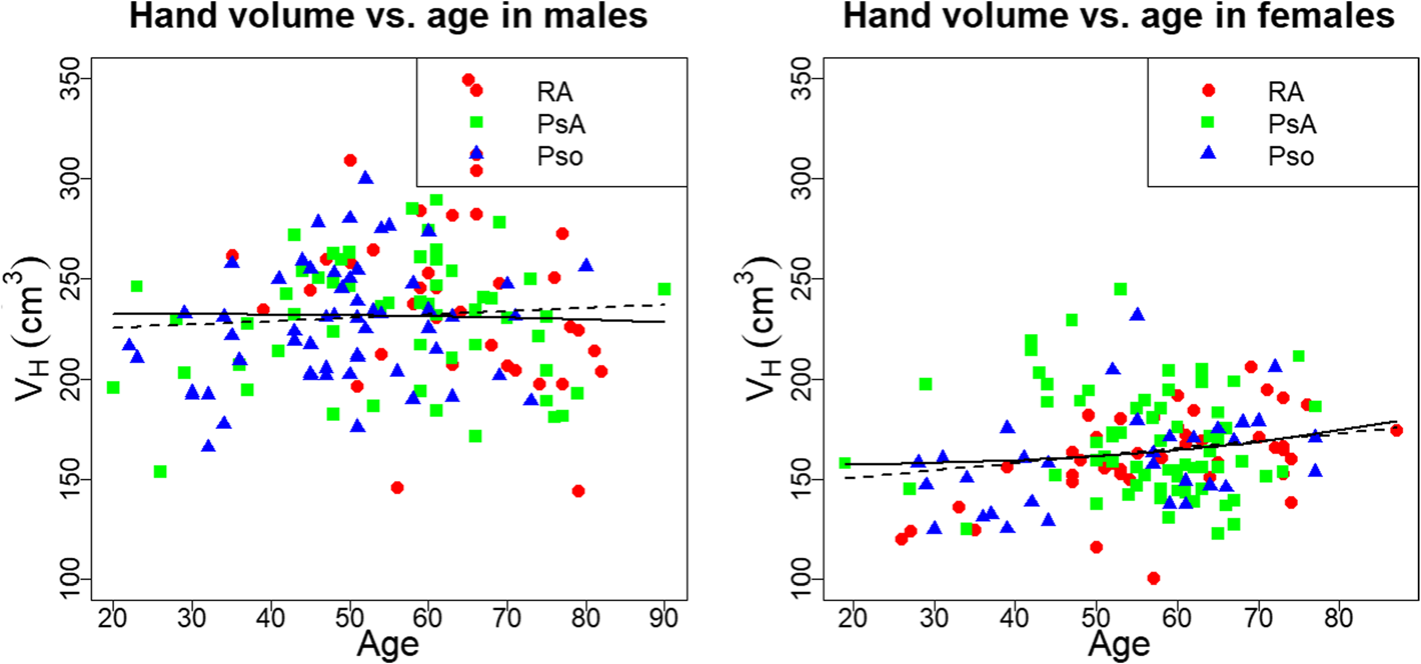
Age dependency of hand volume for males (left) and females (right). Additionally displayed are the regression line of the model linear (solid black line) and cubic in age (dashed black line) for the union of the three groups

Males had a higher hand volume (37% for Pso, 34% for PsA, 48% for RA) than females. The dependence of V_H_ on age is displayed in Fig. 4. In males, V_H_ was correlated positively with age in the Pso (*p* = 0.02) and negatively in the RA group (*p* = 0.05). In females, a positive correlation between V_H_ and age was found for Pso (*p* = 0.01) and RA (*p* < 0.001) patients. For PsA patients there was no significant correlation between V_H_ and age, neither for men nor for women. Between 20 and 80 years, hand volume of males decreased by 17% in RA patients, increased by 9% in Pso patients and did not change in PsA patients. In females hand volume increased by 24% in RA and Pso patients and did not change in PsA patients. After adjustment for age alone or for age and BMI, most group differences in V_H_ were not significant. These results are listed in Table 4, with Δ as the V_H_ difference between the two groups, on average in age between 20 and 80. Adjustment for age^3^ instead of age did not significantly change the results.

**Table 4:** Differences in hand volume between RA, Pso and PsA patients after adjustment for age (left column) and age and BMI (right column) as independent covariates

V_H_ / males	age	age & BMI (S_BMI_)
RA – Pso	*p* = 0.08 Δ = + 14	n.s.
PsA – Pso	n.s.	n.s.
RA – PsA	n.s.	*p* = 0.003 Δ = + 25
V_H_ / females		
RA – Pso	n.s.	n.s.
PsA – Pso	n.s.	*p* = 0.05 Δ = − 0.13
RA – PsA	*p* = 0.07 Δ = − 8.5	n.s.

Δ denotes the V_H_ difference (measured in cm^3^) between two groups, averaged over age. Data after age and BMI adjustment were only obtained in subgroup S_BMI_

### Assessment of relative muscle volume

**Fig. 5:**
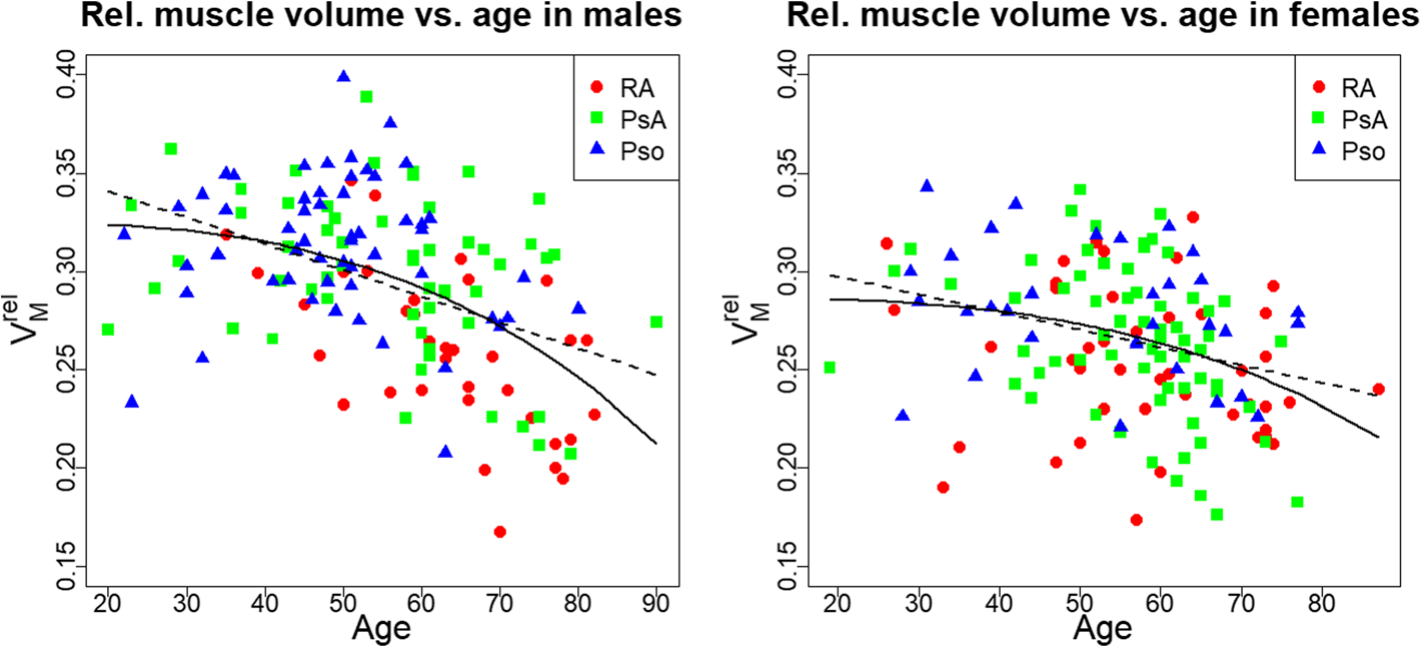
Age dependency of relative muscle volume for males (left) and females (right). Additionally displayed are the regression line of the model linear (solid black line) and cubic in age (dashed black line) for the union of the three groups

Males had a higher relative muscle volume than females (14% for Pso, 11% for PsA, n.s. for RA). The dependence of V_M_^rel^ on age is shown in Fig. 5. The models using age^3^ instead of age better described the age-dependence of V_M_^rel^. When combining data of all three groups, in males the model using age^3^ explained 24% of the variance of V_M_^rel^, compared to 19% for the model linear in age. In females the model using age^3^ explained 11% and the linear one 9% of the variance of V_M_^rel^. However, differences between independent variables age or age^3^ were not observed when comparing the age dependence of V_M_^rel^ among groups. Therefore group differences in V_M_^rel^ will only be reported using models linear in age.

Between 20 and 80 years, negative correlations of relative hand muscle volume with age were observed for male PsA (*p* = 0.01) and RA patients (*p* < 0.001) and for female PsA patients (p < 0.001). Correlations for the other groups were not significant. Male PsA patients lost 16%, male RA patients 27% and female PsA patients 30% of their relative muscle volume. When restricting the analysis to the range between 40 and 80 years, a negative correlation of relative hand volume with age was also observed for male Pso patients (p = 0.01). Between 40 and 80 years male Pso patients lost 13%, male PsA patients 16%, male RA patients 23% and female PsA patients 30% of their relative muscle volume.

Group differences after adjustment for age alone or for age and BMI are shown in Table 5. The Δ stands for the V_M_^rel^ difference between the two groups, on average in the denoted age intervals. Between 20 and 80 years, relative muscle volume in males RA patients was 16% lower and in females 9% lower than in Pso patients. In males but not in females there was also a significant difference between RA and PsA patients, relative muscle volume was 13% lower in RA patients.

**Table 5:** Differences in relative muscle volume between RA, Pso and PsA patients after adjustment for age and age and BMI as independent covariates

	Age 20–80 years		Age 40–80 years	
V_M_^rel^ / males	age	age & BMI (S_BMI_)	age	age & BMI (S_BMI_)
RA – Pso	*p* < 0.0001 Δ = − 0.040	*p* < 0.001 Δ = − 0.045	*p* < 0.0001 Δ = − 0.041	*p* < 0.0001 Δ = − 0.039
PsA – Pso	n.s.	*p* = 0.05Δ = − 0.021	n.s.	n.s.
RA – PsA	*p* < 0.0001 Δ = − 0.033	*p* = 0.02 Δ = − 0.023	*p* < 0.0001 Δ = − 0.045	*p* = 0.02 Δ = − 0.024
V_M_^rel^ / females				
RA – Pso	*p* = 0.01 Δ = − 0.023	*p* = 0.09 Δ = + 0.020	*p* = 0.03 Δ = − 0.022	*p* = 0.03 Δ = − 0.020
PsA – Pso	n.s.	n.s.	n.s.	*p* = 0.05 Δ = − 0.019
RA – PsA	n.s.	n.s.	n.s.	n.s.

Δ denotes the V_M_^rel^ difference between two groups, on average in the denoted age interval. V_M_^rel^ ranges between 0 and 1, thus Δ*100 stands for the percentage point difference of relative muscle volume. Data after age and BMI adjustment were only obtained in subgroup S_BMI_. The calculations are repeated for the age group 40–80 years (two most-right columns)

BMI alone did not show significant correlations with V_M_^rel^ in either sex. However, after additional adjustment for BMI, in females the difference V_M_^rel^ between RA and Pso patients was no longer significant, while in males the difference between PsA and Pso patients became significant (*p* = 0.05).

Results were similar in the age group 40–80 years but in males there was no significant difference, between PsA and Pso patients even after adjustment for BMI, while the difference became significant in females.

**Fig. 6:**
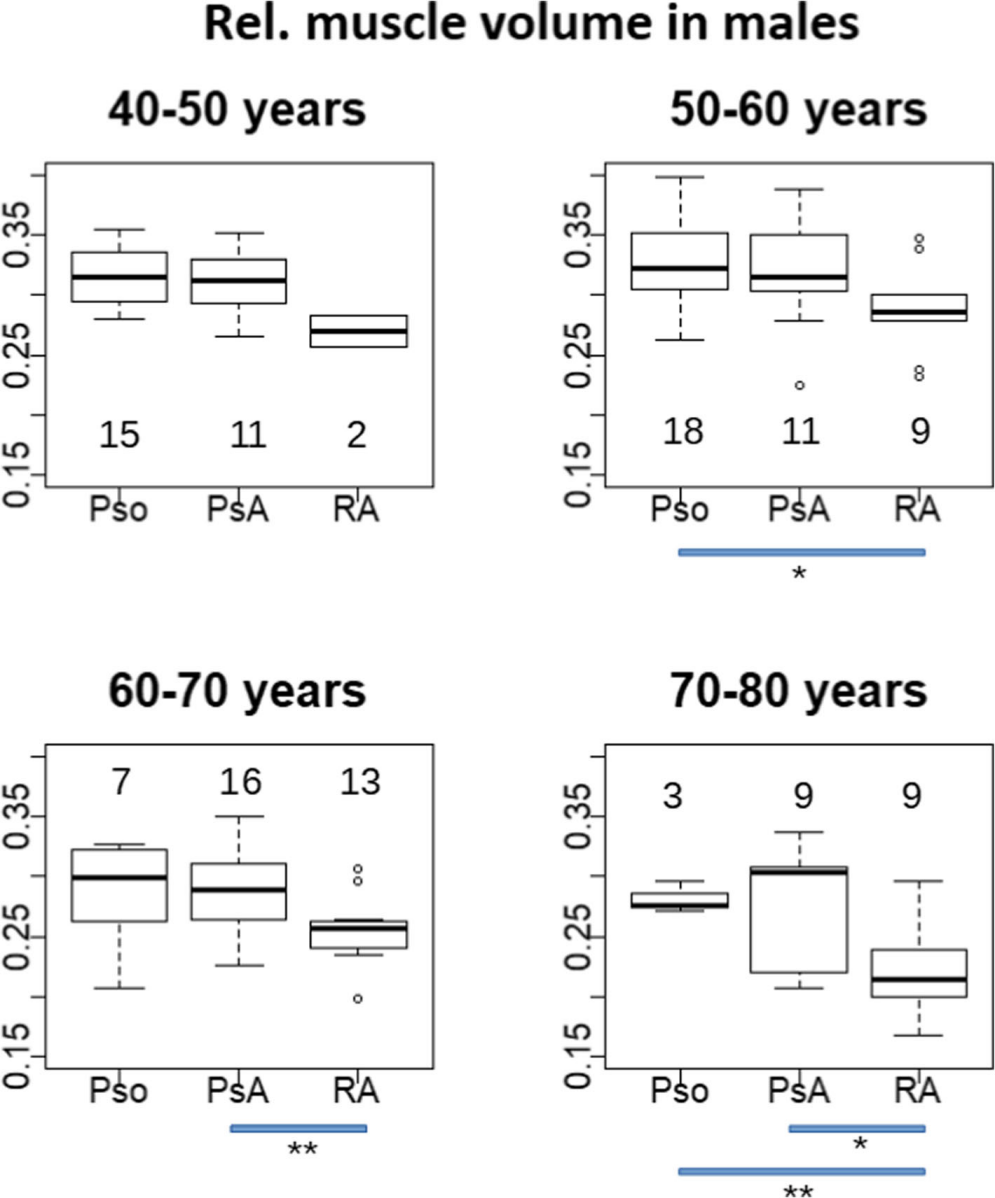
Relative muscle volume displayed for the four decades between 40 and 80 years for females. The number of patients (n) per box are displayed above/below them. The blue bars below each panel are the significance level of the corresponding group differences, with * (*p* < 0.05), ** (*p* < 0.001) and *** (*p* < 0.0001)

**Fig. 7:**
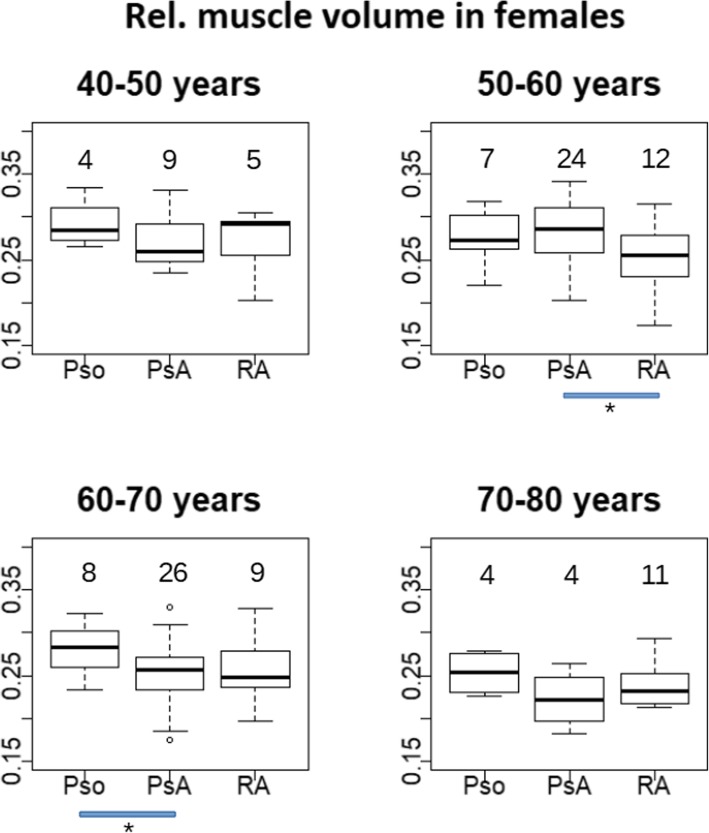
Relative muscle volume displayed for the four decades between 40 and 80 years for males. For the description of the numbers and blue bars see Fig. 6

Results of V_M_^rel^ by age decade are shown in Fig. 6 for males and Fig. 7 for females. For males, there was a consistent pattern of numerically lower relative hand muscle volume in RA patients for all decades. Significant differences of V_M_^rel^ among groups in males were observed for the 5th decade (RA-Pso, *p* = 0.04, mean difference = − 0.046), 6th decade (RA-Pso, p = 0.04, mean difference = − 0.039) and 7th decade (RA-PsA, p = 0.04, mean difference = − 0.03) but not for the 8th decade. In females, group differences were not significant for any decade.

### Assessment of muscle fat

Absolute intramuscular fat volume (V_F_) was positively correlated with hand volume V_H_ (R^2^ = 0.74, *p* < 0.0001). Age, disease and BMI did not significantly impact the correlation.

**Fig. 8:**
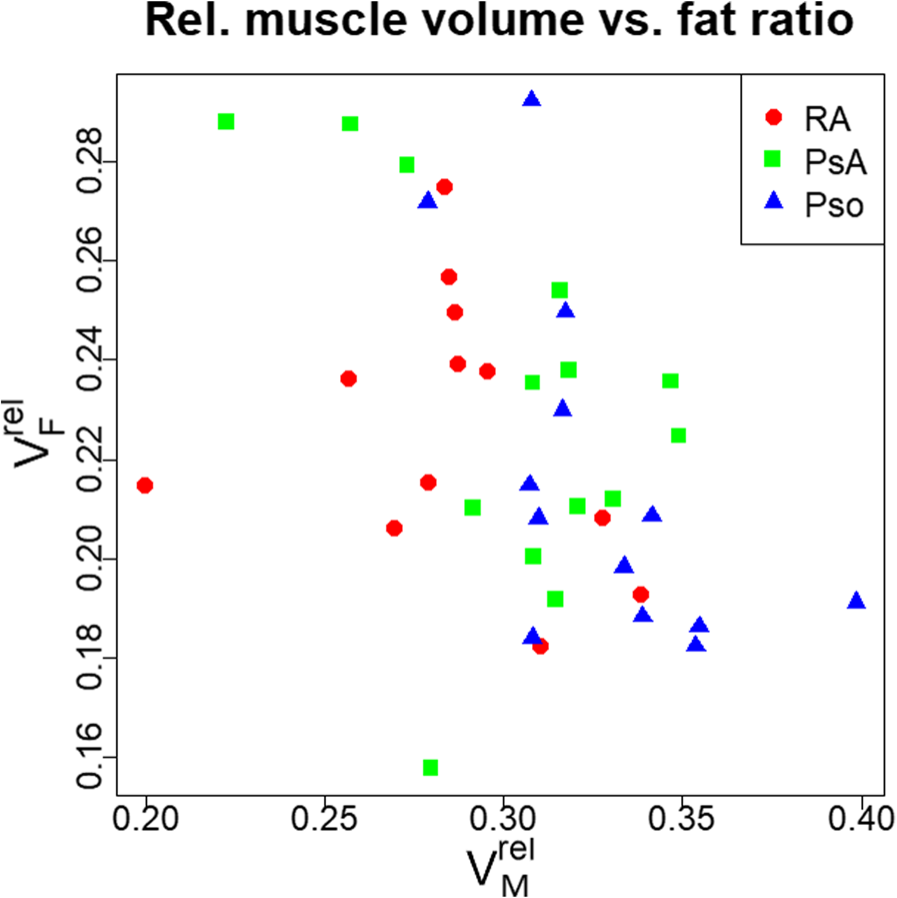
Fat ratio plotted against relative muscle volume for all groups and both sex

Relative intramuscular fat content (V_F_^rel^) showed no significant correlation with hand volume but a significant negative correlation with V_M_^rel^ (R^2^ = 0.18, *p* = 0.004) (Fig. 8).

## Discussion

In this study we compared hand muscle volume in patients with Pso, PsA and RA. For the purpose of this study subjects with Pso were regarded as control group as Pso patients show only little to no inflammation in the hand joints.

We applied a combination of T1-weighted and 2 pt-Dixon imaging sequences and a random forest based segmentation method to analyze muscle volume. Hand muscle fat infiltration could only be obtained in a small subset so far. The excellent precision of MR 2 pt-Dixon for fat quantification had been demonstrated earlier [24]. In the same study a high correlation of fat content between 2 pt-Dixon measurements of phantoms and MR spectroscopy, the standard method for fat quantification was reported.

The central findings of this study showed that the age related decrease of relative hand muscle volume in males was larger than in females (Table 5 and Figs. 6 and 7) and that in males but not in females relative muscle volume was lower in RA than in PsA or Pso patients, independent of age. Results did not significantly change when substituting age with age^3^.

Not surprisingly, males had a higher hand volume than females. Gender differences of relative muscle volume were smaller but with the exception of subjects with RA, males also had higher relative muscle volume than females. In the control (Pso) group, age related decreases were larger for relative muscle than for hand volume, which can be explained by increasing muscle fat infiltration with age. Our very preliminary and still limited results, which showed an inverse relation between relative fat and relative muscle volume support this hypothesis.

A detailed investigation of age related changes per disease group and age decade showed that in the male control group decreases in relative muscle volume started in the sixth decade, indicating progressive sarcopenia. There were no significant difference between the control group and PsA patients for either decade. In males with RA, V_M_^rel^ was lower than for the two other groups for all age decades and the decline of V_M_^rel^ was larger, in particular in the 8th decade. This decrease in V_M_^rel^ is further amplified by the age related decrease in hand volume showing the strong association between muscle deterioration and RA in male subjects. Differences in relative muscle volume among Pso, PsA and RA female patients were numerically smaller and not significant for most age decades.

The results shown in Figs. 6 and 7 were confirmed after age adjustment in the pooled age groups (Table 5). In males and females, differences in V_M_^rel^ were significant between RA and Pso patients but age adjusted differences were about twice as high in males. It is speculative but perhaps this larger difference in V_M_^rel^ between male RA patients and controls could explain why in males but not in females the difference between RA and PsA groups was also significant. In other words, the main difference in V_M_^rel^ between males and females is observed in the RA group. Whether this can be explained by the degree of inflammation, by differential therapeutic interventions, differences in the efficacy of an intervention or the way the hands are used in daily life is an interesting questions that warrants further investigation. Unfortunately the power of our data was insufficient to further explore the differential contribution of intramuscular adipose tissue in male and female RA patients.

As already indicated in the background section, most RA patients have a normal BMI. This fact was indirectly confirmed in this study. No or only very small, mostly non-significant effects of BMI were observed on V_H_, V_M_^rel^ and V_F_^rel^. Probably the hand, which was the focus of this study is too small in order to show a significant impact on BMI, which is a whole body parameter. However, BMI also did not reflect changes in fat content caused by fatty infiltration in paraspinal [25], or gluteus muscles [26].

This study was a pilot study to demonstrate that MRI can be used to assess hand and hand muscle volume and intramuscular adipose tissue and to demonstrate differences between patients with Pso, PsA and RA. There are a number of limitations that should be addressed in future investigations:

First, the use of Pso patients as controls could be criticized as recently subclinical inflammation has been reported in Pso patients [15]. Thus a control group of healthy subjects should also be investigated. Second, functional measurements such as grip strength, data on pharmaceutical interventions and other diagnostic scores should be added as covariates to the analysis. Third, fat measurements should be generated in a larger number of subjects. Fourth, this was a cross-sectional study, correlations with age need cautious interpretations. Longitudinal data still have to be collected.

## Conclusions

These preliminary data showed that relative hand muscle volume decreased with age in male and female patients with Pso, PsA and RA patients. Independent of age, relative hand muscle volume was smaller in patients with RA compared to the patients with Pso and the difference was twice as large in males compared to females. Also in male but not in female RA patients relative hand muscle volume was smaller than in PsA patients. Preliminary results further indicate that the decrease in relative hand muscle volume is associated with an increase of relative fat content.

## Data Availability

The datasets generated and/or analysed during the current study are not publicly available since the consent does not include an agreement to put the patient images in public domain, but are available from the corresponding author on reasonable request.

## Abbreviations

BMI: Body mass index
CSA: Cross sectional area
I_fat_: Dixon fat image
I_ff_: Dixon fat fraction image
I_water_: Dixon water image
MCP: Metacarpal bone
MRI: Magnetic resonance imaging
PsA: Psoriatic arthritis
Pso: Psoriasis
RA: Rheumatoid arthritis
RC: Rheumatoid cachexia
S_BMI_: Subgroup of patients with available BMI data
S_dixon_: Subgroup of patients with available MR dixon images
TSE: Turbo spin echo
V_F_: Fat volume
V_F_^rel^: Relative fat volume
V_H_: Hand volume
V_M_: Muscle volume
V_M_^rel^: Relative muscle volume (= V_M_ / V_H_)
VOI: Volume of interest
